# Supplementary material for: Genetic evaluation of growth rate and efficiency-related traits in Menz sheep
Source: PLoS One. 2025 Dec 3;20(12):e0337718. doi: 10.1371/journal.pone.0337718 (PMC12674582; doi:10.1371/journal.pone.0337718)
Supplement: Attachment — Submitted filename: Kebede_comment_Menz.docx [file pone.0337718.s002.docx]

Thanks for inviting manuscript number PONE-D-25-45437: Genetic evaluation of growth rate and efficiency-related traits in Menz sheep. I try to review comments, and I will give detailed below

**Title**

consider shortening to “Genetic parameters of growth and efficiency traits in Menz sheep” for conciseness. I also saw an article on Dorper at the same research center by same author

- “Efficiency-related traits” could be specified (e.g., Kleiber ratio, relative growth rate). Kleiber is not commonly used. Put research gap

**Abstract**

- L14–16: The phrase *“traits related to growth efficiency are economically important and genetic improvement of these traits is vital”* is slightly repetitive. Suggest: *“Growth efficiency traits are economically important, and their genetic improvement is essential.”*
- L17–22: The abstract lists too many traits (6MW, ADG1, ADG2, ADG3, KR1, etc.) without context. This overwhelms the reader. Consider grouping (e.g., “average daily gain, Kleiber ratios, efficiency of growth, and relative growth rate”).
- L29–32: *“Direct additive genetic effect as the unique known random effect was the best fitting model …”* → grammatically awkward. Suggest: *“The model with direct additive genetic effect alone provided the best fit for most traits, except 6MW and ADG1, where maternal effects were also important.”*
- L33–37: Heritability estimates are listed as a long series of numbers. Better to summarize ranges (e.g., *“heritability ranged from 0.21 to 0.48 across traits”*).
- L42–44: *“Selection for 6MW would be more effective …”* → Please clarify why six-month weight is the most informative trait. Already published so many times, repeated finding. Think new idea, a new suggestion that can advance the ongoing CBBP

**Keywords**

- Good selection, but you may add **“Menz sheep”** explicitly as a keyword to improve discoverability.

**Introduction**

- L49–56: Good background, but sentence *“Small ruminants have been significantly supporting the livelihoods of smallholder farmers…”* is awkward. Suggest: *“Small ruminants play a major role in supporting the livelihoods of smallholder farmers across Ethiopia’s diverse ecological zones.”*
- L57–66: The problem statement (low productivity despite adaptation) is strong. However, you need to clearly define the **research gap**: Few studies have estimated genetic parameters for efficiency traits (Kleiber ratio, RGR, GE) in Menz sheep.
- L67–72: The introduction to Kleiber ratio is appropriate, but the rationale for including efficiency traits should be emphasized. Add supporting references from similar indigenous sheep studies.
- Kleiber? I did not understand its importance
- L73–78: The objective statement is clear, but could be sharper: *“This study aimed to estimate genetic parameters for growth rate and efficiency-related traits in Menz sheep under community-based breeding programs.”*

**Materials and Methods**

- **Location & flock description (L80–96):**
  - Good detail about geography and climate. However, some redundancy (adaptability of Menz sheep already mentioned in Introduction).
  - Suggest summarizing climatic info in fewer words, and moving references on adaptability to Introduction.
- **Data collection (L97–111):**
  - L97: *“New born lambs were weighted”* → “weighed.”
  - L105: *“…primarily focused on using six months weight as selection criteria”* → “as the main selection criterion.” Since already described by other author just cite the previous authors. The procedure of CBBP selection is as described by ----------
  - L109–111: Provide clarity: *“Rams were rotated annually between groups to minimize inbreeding.” Still duplication*
- **Dataset (L112–126):**
  - Excellent that you specify sample size (22,712 records, 6,528 ewes).
  - L117–118: Typo: ADG1 repeated twice (birth–weaning and weaning–6 months). One should be ADG2.
- **Trait definitions (L127–137):**
  - Equations should be formatted clearly with subscripts and consistent notation.
  - L133: Efficiency of growth formula is confusing; add units or explanation (percentage change relative to initial weight).
- **Statistical models (L138–192):**
  - L172: *“…when the change in maximum log L between the last two iterations is less than 10-4”* → please write as 1e-4 for clarity.
  - Genetic trend analysis (L190–192): Provide more detail on the time scale (2009–2023). Were all traits analyzed for trends or only key ones? Need clarification

**Results**

- L193–207: Environmental effects are described well. However:
  - Phrase *“pedigree structures utilized in this study are fair enough to accurate genetic parameters”* → awkward. Suggest: *“The pedigree structure was adequate for accurate estimation of genetic parameters.”*
- L208–217: Heritability values should again be summarized in ranges, with key examples. The full list can stay in the table.
- L218–229: Correlations are strong, but the text is hard to follow. Suggest breaking into sub-sections: (i) within-period correlations, (ii) across-period correlations.
- Figures 1–3: Ensure captions explain the units of genetic trend (kg/year, g/day/year, etc.).

**Discussion**

- L231–239: Good explanation of environmental effects. Could improve by citing more Ethiopian sheep literature beyond Baluchi/Kermani references.
- L243–246: The hormonal explanation for sex differences is good, but speculative. Please cite a specific physiological reference.
- L248–252: The finding that twins were more efficient pre-weaning but less efficient post-weaning is very interesting. This needs stronger discussion and reference support.
- L260–283: Discussion on heritability estimates is strong. However, some sentences are too long and repetitive. Simplify.
- L285–307: The point about unfavorable pre- vs. post-weaning correlations is very important. Please emphasize its implication: selection should consider multi-trait indices to avoid negative correlated responses.

**Conclusion**

- L315–329: Well-structured, but slightly repetitive. Suggest trimming.
- Add practical recommendation: e.g., “Breeding programs should prioritize six-month weight while monitoring efficiency-related traits to ensure balanced progress.” Too many times documented- see previous article including Bonga, Doyogena

**References**

- Generally good coverage, including Ethiopian studies.
- Some inconsistencies:
  - L366: “and a pastoral system of ethiopia” → capitalize Ethiopia.
  - Several references (e.g., #5) are poorly formatted and need editing.
- Please ensure consistent journal abbreviations (Small Ruminant Res. vs. Small Rumin Res.).

**Tables & Figures**

- Tables are informative but crowded. Suggest splitting if possible.
- Use consistent superscript letters for multiple comparisons (currently a, b, c, but sometimes overlapping).
- Figures of genetic trends should include error bars or confidence intervals.
